# Seasonal changes in the digesta-adherent rumen bacterial communities of dairy cattle grazing pasture

**DOI:** 10.1371/journal.pone.0173819

**Published:** 2017-03-15

**Authors:** Samantha J. Noel, Graeme T. Attwood, Jasna Rakonjac, Christina D. Moon, Garry C. Waghorn, Peter H. Janssen

**Affiliations:** 1 Grasslands Research Centre, AgResearch Limited, Palmerston North, New Zealand; 2 Institute of Fundamental Sciences, Massey University, Palmerston North, New Zealand; 3 Department of Animal Science, Aarhus University, Tjele, Denmark; 4 DairyNZ, Hamilton, New Zealand; Wageningen University, NETHERLANDS

## Abstract

The complex microbiota that resides within the rumen is responsible for the break-down of plant fibre. The bacteria that attach to ingested plant matter within the rumen are thought to be responsible for initial fibre degradation. Most studies examining the ecology of this important microbiome only offer a ‘snapshot’ in time. We monitored the diversity of rumen bacteria in four New Zealand dairy cows, grazing a rye-grass and clover pasture over five consecutive seasons, using high throughput pyrosequencing of bacterial 16S rRNA genes. We chose to focus on the digesta-adherent bacterial community to learn more about the stability of this community over time. 16S rRNA gene sequencing showed a high level of bacterial diversity, totalling 1539 operational taxonomic units (OTUs, grouped at 96% sequence similarity) across all samples, and ranging from 653 to 926 OTUs per individual sample. The nutritive composition of the pasture changed with the seasons as did the production phase of the animals. Sequence analysis showed that, overall, the bacterial communities were broadly similar between the individual animals. The adherent bacterial community was strongly dominated by members of Firmicutes (82.1%), followed by Bacteroidetes (11.8%). This community differed between the seasons, returning to close to that observed in the same season one year later. These seasonal differences were only small, but were statistically significant (*p* < 0.001), and were probably due to the seasonal differences in the diet. These results demonstrate a general invariability of the ruminal bacterial community structure in these grazing dairy cattle.

## Introduction

The rumen is a specialised organ that allows fermentation of ingested plant material by a complex consortium of microbes including prokaryotic bacteria and archaea, and eukaryotic fungi and protozoa. The rumen microbiota converts ingested plant matter to microbial protein and volatile fatty acids (VFA), which provide ~70–85% of the nutrients absorbed by the ruminant [1, 2]. Significantly, plant polysaccharides that are unable to be degraded by the mammalian host can be fermented by rumen microbes to products that the ruminant can use. The types of fermentation products and the rates at which they are produced in the rumen regulate the composition and yield of the main commercial products from farmed ruminants, namely milk, meat, and wool [3]. Since the combined actions of the rumen microbes are responsible for feed fermentation, it is important to understand these communities to gain a better understanding of rumen function and its impact on the health and productivity of the animal.

Bacteria are the most numerous and diverse group of the rumen microbes. The bacteria that attach to ingested plant matter within the rumen are thought to be responsible for initial fibre degradation [4, 5]. The bacterial species that adhere to ingested feed are reported to be different from the planktonic bacteria in the rumen fluid and those associated with the rumen epithelium [6–8]. Recently, the digesta-adherent community has been specifically examined because of the potential to learn more about the process of fibre degradation in the rumen [6, 9]. A greater understanding of the interactions and dynamics of bacteria and the feed could lead to improvements in ruminant feed use, fibre degradation, and fermentation efficiency. This includes understanding which bacteria are responsible for degradation of different feed components.

The best-studied and perhaps the most important factor influencing the rumen bacterial community structure is the host diet, as it determines the substrates available for rumen fermentation [6, 10–13]. The stage in the production cycle of cattle strongly affects the nutritional needs of the animals and hence their feeding patterns. Other factors that can influence bacterial community structure include geographic area [14], ruminant species [15], animal-to-animal variation [16], rumen developmental status [17, 18], lactation [8], photoperiod [19] and heat stress [20, 21]. Although changes in the host diet and environmental conditions have been shown to influence the microbial community composition, little is known about the bacterial community changes over time when ruminants are on the same or similar diets. Because different animals of the same species fed the same diet can exhibit divergent rumen bacterial communities [22, 23], it is necessary to follow the rumen community of the same animal to systematically examine the effect of time and season.

The use of phylogenetically-informative marker genes, such as the 16S rRNA gene, to describe rumen microbial communities allows a comprehensive depiction as it includes the large percentage of uncultured and undescribed organisms present in the rumen. In this study, the digesta-adherent rumen community composition of individual farmed New Zealand dairy cows, maintained under production conditions, was monitored by sampling in each season over the course of a year, with the last sample taken in the same season as the first but a year later. It is standard practice for New Zealand dairy cattle to be farmed with a seasonal system, meaning the cows calve at the same time on a yearly cycle and the whole herd is at the same phase in the production cycle. The quality of pasture feed available in each season was expected to differ [24] as was the phase in the production cycle and the feed was supplemented with pasture silage in autumn and winter in accordance with normal farming practice, potentially affecting the rumen community composition. Therefore, examining the same animals over different seasons could capture greater ruminal bacterial diversity and offer the opportunity to examine the stability of the bacterial community under production conditions. Pyrosequencing of the V1-V3 region of the bacterial 16S rRNA gene was used to provide higher resolution on the variation of the digesta-adherent bacterial community in individual animals and at different sampling times.

## Materials and methods

### Animal handling and sample fractionation

Use of the animals for rumen sampling was approved by the AgResearch Ruakura Animal Ethics Committee under permit number AE 11483 and was conducted in accordance with the institutional Codes of Ethical Conduct for the Use of Animals in Research, Testing and Teaching, as prescribed in the New Zealand Animal Welfare Act of 1999 and its amendments. Rumen samples from five ruminally fistulated Holstein/Friesian cows (four animals at each time point) that were part of a normal dairy herd were taken at five time points over the course of a year. One animal (Cow A) sampled in the first sample season (May) subsequently died and was replaced by a fifth animal (Cow E) for the remainder of the experiment. Cows calved in late winter (July/August) and samples were taken during the dry period (not lactating) in autumn (May, **M**) and during lactation in winter (August, **A**), spring (November, **N**), summer (February, **F**), and then again in autumn (dry period) one year after the sampling started (May +1yr, **L**). The animals were selected to represent those in a New Zealand dairy herd in regards to age and milk production (Table 1) and were maintained as part of a larger single herd at Scott farm, Dairy NZ, Vaile Road, Hamilton, New Zealand. The herd grazed *ad libitum* on a rye-grass/clover dominant pasture, and the feed was supplemented with pasture silage made from the same pasture type as the grazing pasture during the months when feed was limited, and this included both May and the August sampling times. The amount of silage offered was around 30% of the recommended daily intake during these months. Rumen grab samples of total content (1.5 kg) were taken via the fistula at about 07.30 h (after morning milking and before a new allocation of feed was given) from several locations within the rumen and transported to the laboratory in a CO_2_-flushed container, where the pH was measured and samples were processed immediately.

**Table 1:** Milk production over the lactation during which the cows were sampled.

Cow	Age in years	Lactation length (days)	Annual milk (kg)	Milk protein (kg)	Milk fat (kg)
B	5.7	271	5156	179.6	228.7
C	4.7	289	4758	195.3	237.8
D	4.7	297	5975	207.0	245.0
E	5.8	266	5829	203.3	261.6

An optimised protocol for separating digesta-adherent bacteria from planktonic and loosely attached bacteria was used to fractionate the rumen samples. All procedures were performed under a continuous flow of CO_2_ gas to maintain anaerobic conditions. Briefly, 200 g of rumen contents was removed from the centre of the collected rumen sample and squeezed through a double layer of cheesecloth with a mesh size of approximately 1 mm (Stockinette Cirtex Industries Ltd., Thames, New Zealand) to obtain digesta solids. A fraction (50 g) of these digesta solids was washed four times by suspending it in 200 ml of anaerobic RM02 base media [25], followed by straining through a double-layer of cheesecloth. A fraction of digesta that had been washed four times (exactly 40 g) was mixed with 360 ml of anaerobic RM02 and then disrupted in a Waring blender (Waring Products Inc., Torrington, CT, USA) for four 20-s pulses with a 30-s pause between each pulse to produce the digesta-adherent fraction. All fractionated samples were immediately frozen and stored at −85°C and the digesta-adherent fraction was used for later DNA extraction.

### Feed analyses

Pasture samples were collected by hand from the pasture the animals were grazing the day prior to each sampling day, to represent the material eaten. Pasture and silage analysis was carried out by a commercial feed analysis service (feedTECH, Palmerston North, New Zealand). The samples were dried to constant weight at 65°C and then ground to a particle size that could pass through a 1-mm sieve. Near-infrared reflectance spectroscopy (NIRS) was used to estimate crude protein (CP), crude fat (lipid), ash, acid detergent fibre (ADF), neutral detergent fibre (NDF), reducing sugars—soluble sugars and starch (SSS), organic matter digestibility (OMD), and metabolisable energy (ME) content, expressed on a dry matter basis, as described by Corson *et al*. [26].

### DNA extraction

Rumen samples were stored at −80°C, then lyophilised in a freeze drier for 3 days until completely dry (Cuddon, Blenheim, New Zealand), followed by grinding to a fine powder in a clean coffee grinder (Russell Hobbs, Mordialloc, Vic., Australia). DNA was extracted using a phenol-chloroform extraction protocol including bead beating (Henderson *et al*., 2013). Briefly, about 30 mg of freeze-dried rumen sample was weighed into a 2-ml screw-cap tube containing 0.7 g of pre-baked 0.1-mm zirconium beads (Biospec Products, USA). The following was then added: 450 μl of 120 mM sodium phosphate buffer (112.87 mM Na_2_HPO_4_, 7.12 mM NaH_2_PO_4_, pH 8.0; filter-sterilized through a 0.2-μm pore size filter and autoclaved), 150 μl of TNS solution (500 mM Tris-HCl, pH 8.0, 100 mM NaCl, 10% sodium dodecyl sulfate [w/v]; filter-sterilized through a 0.2-μm pore filter, then autoclaved) and 600 μL of phenol/chloroform/isoamyl alcohol (25:24:1, pH 8.0). Samples were disrupted in a BIO101 Fast-Prep FP120 cell disrupter (Thermo Savant, Carlsbad, CA, USA) for 45 s (6.5 m/s) and immediately placed on ice. Tubes were then centrifuged at 13,000 × g at 4°C for 20 min.

The aqueous phase (top layer; 400 μL) was transferred to a 2-ml tube and extracted with 400 μL of chloroform/isoamyl alcohol (24:1) solution by inverting the tube several times, followed by centrifugation at 13,000 × g at 4°C for 5 min. The aqueous phase (top layer; 350 μL) was transferred to a fresh tube and mixed with 700 μL of PEG solution (30% [w/v] polyethylene glycol 6000 in 1.6 M NaCl, prepared with RNAse free water, then autoclaved) to precipitate the DNA. The solution was mixed by carefully inverting the tube 5 times and DNA was pelleted by centrifugation at 13,000 × g at 4°C for 60 min. The supernatant was carefully removed and the pellet washed twice with 500 μL of chilled 70% ethanol and centrifuged again for 10 min at 13,000 × g at 4°C. The ethanol was carefully removed from the DNA pellet by pipetting and the pellet dried at 50°C for at least 15 min.

The dried purified DNA was dissolved in 200 μL of EB buffer (10 mM Tris-HCl, pH 8.5, prepared with RNAse free water and filter-sterilized through a 0.2 μm pore size filter then autoclaved) at 4°C overnight. DNA concentrations were measured by fluorometry using the Quant-iT dsDNA Broad-range Assay kit (Life Technologies, Carlsbad, CA, USA). Purified DNA samples were stored at −20°C and diluted to 4 ng/μL with 10 mM Tris-HCl, pH 8.5, before use.

### Pyrosequencing and data analysis

The V1-V3 region of the bacterial 16S rRNA gene was amplified from each DNA sample using a conserved primer pair; 27f [27] and 514r [28] with a unique 10-nucleotide barcode (MID sequence) at the 5'-end of the forward primer (S1 Table). PCR was performed in a 50 μL reaction mixture containing a HiFi platinum Taq (0.02 U/μL; Life Technologies), 1 × HiFi PCR buffer (Life Technologies), 2 mM MgSO_4_, 0.2 mM of each dNTP, 0.4 pmol/μL of each primer and 0.08 ng/μL of template DNA. The PCR program was as follows: initial denaturation at 94°C for 2 min, followed by 35 cycles (94°C for 30 s, 56°C for 30 s and 68°C for 45 sec). The final elongation was at 68°C for 20 min. A negative control (no template DNA) using the forward primer with a unique barcode (MID sequence) was set up and processed alongside the other samples. To investigate any primer effects, one sample was amplified twice, each with a different barcoded (MID sequence) primer (S1 Table). All samples including the no-template control and the duplicate sample were amplified in triplicate, with each replicate in a separate run on the thermocycler, and the PCR products were pooled by sample. The pooled individual samples were analysed by agarose gel electrophoresis, stained with ethidium bromide and viewed using UV transillumination. The gel image was captured with a Gel Logic 200 imaging system and quantified from the gel image using Kodak gel doc software (Carestream Health, New Haven, CT). An equivalent amount of each sample was pooled together, and the total pooled mix from all 22 samples subjected to preparative agarose gel electrophoresis (1.1% [w/v] agarose) pre-stained with 2 × SYBR safe. A single band (~500 bp) was visualised on a Safe Imager 2.0 Blue-Light Transilluminator (Invitrogen, Carlsbad, CA, USA), excised, and the DNA was extracted from the gel slices using the Wizard SV Gel and PCR Clean-Up System (Promega, Madison, WI, USA) following the manufacturer’s instructions. Barcoded pyrosequencing was carried out by Macrogen Inc. (Seoul, Korea) using a 454 Genome Sequencer FLX Instrument and GS FLX titanium chemistry (454 GS FLX; Roche 454 Life Sciences, Branford, CT, USA). Sequences have been deposited in NCBI under SRA accession SRP067626. Pre-processing and quality control of the reads were performed using the QIIME pipeline [29, 30]. The pyrosequence reads underwent further quality control and clustering analysis using CD-HIT-OTU [31], which removed noise and chimeric reads and then clustered the denoised reads in the following steps. (i) Low-quality reads were filtered out and reads were trimmed to the expected size. No reads were removed in this step because they had already been quality-checked in QIIME. (ii) Filtered reads were clustered at 100% identity using CD-HIT-DUP. (iii) Chimeric clusters were identified and removed. (iv) Secondary clusters were identified and recruited into primary clusters. (v) Noise sequences were designated as those in clusters of 1 or 2 reads. (vi) The remaining processed dataset from non-chimeric clusters was reclustered into OTUs at 96% similarity level.

Representative sequences from each OTU were aligned using PyNAST [32]. A taxonomic identity was assigned to each representative sequence using UCLUST [33] and the greengenes database for reference (release gg_13_8_otus, downloaded from http://greengenes.lbl.gov). The alignments were filtered before being used to create a phylogenetic tree using the fasttree method in QIIME [34]. Further analysis was performed using the tools available in QIIME version 1.8.0. A taxonomy summary chart and tables were derived from the OTU table, allowing visualisation of the overall bacterial community structure in each of the samples. Rarefied diversity matrices were calculated (sample depth = 4607) to compare the types of communities based on the taxonomic assignments. The differences were visualised using UniFrac analysis and Principal Coordinate Analysis (PCoA) plots in R (www.r-project.org). The non-parametric permutational MANOVA-based statistical tests adonis and ANOSIM [35] were implemented in QIIME using the compare_categories.py script. Animal variation was examined with the following linear mixed model created in R with the nlme package [36] using summarised weighted and unweighted UniFrac distances for each sample [37].

$$Y_{ijk}=\mu +S_{i}+a_{j}+e_{ijk}$$

where *Y*_*ijk*_ the *k*th measurement of the *i*th season on the *j*th animal defined as the combined pairwise dissimilarity as captured by the weighted or unweighted Unifrac dissimilarity matrix, *μ* is the grand mean, *S*_*i*_ is the effect of the *i*th season (i = 5; *a*_*j*_ is the random effect of the *j*th animal which is assumed to be normally distributed with a mean on zero and a variance of $\sigma_{a}^{2}$, and *e*_*ijk*_ is the random residual of the *i*th season on the *j*th animal of the *k*th measurement assumed to be normally distributed with a mean of zero and a variance of $\sigma_{e}^{2}$.

The repeatability coefficient is defined as the quotient of the animal variance and the product of the animal and residual variances estimated in the model above ($\frac{\sigma_{a}^{2}}{\left(\sigma_{a}^{2}+\;\sigma_{e}^{2}\right)}$)

## Results

### Rumen and forage nutritive properties

Rumen contents were sampled from cows in a production dairy herd over a 12-month period, in autumn (May), winter (August), spring (November), summer (February), and then again in autumn (May), one year after the sampling started. At each sampling time, the compositions of the pasture and pasture silage that the animals were feeding on were determined (Table 2), the ruminal volatile fatty acid (VFA) concentrations and pH measured, and a description of the rumen contents from each cow recorded (Table 3).

**Table 2:** Nutritive value and components of pasture silage (silage) and fresh pasture (pasture) used as feed for the animals in this study.

Date	Feed	Metabolisable energy (MJ/kg of dry matter)	Organic matter^a^	Acid detergent fibre^a^	Neutral detergent fibre^a^	Crude protein^a^	Crude fat^a^	Organic matter digestibility^a^	NH_4_-N^a^^b^	pH^c^
May	Silage	11.7	90.2	34.0	49.9	16.2	3.8	72.8	9.4	3.9
Aug	Silage	11.6	89.0	35.8	47.2	18.5	3.6	72.6	7.2	4.1
May +1yr	Silage	10.3	88.9	37.9	50.5	14.1	2.8	64.3	7.0	4.5
May	Pasture	11.9	90.9	22.8	38.8	22.9	2.9	82.5	n.d.	n.d.
Aug	Pasture	12.5^d^	88.2	21.3	39.0	29.6	3.7	85.0^e^	n.d.	n.d.
Nov	Pasture	11.7	92.6	27.4	50.8	15.6	2.2	78.2	n.d.	n.d.
Feb	Pasture	11.8	90.0	23.5	43.6	24.2	2.5	80.4	n.d.	n.d.
May +1yr	Pasture	12.5^d^	89.4	21.4	36.4	26.9	3.4	85.0^e^	n.d.	n.d.

n.d. not determined.

^a^Components estimated by NIRS analysis are reported as percent of dry matter.

^b^NH_4_-N, ammonia nitrogen as a fraction of total nitrogen.

^c^pH estimated by NIRS.

^d^NIRS estimates are limited to a ME 12.5 MJ/kg of DM.

^e^NIRS estimates are limited to 85% organic matter digestibility.

**Table 3:** Characteristics of rumen contents of cows sampled over the course of a year.

Date	Cow	Sample code	Succinic acid^a^	Lactic acid^a^	Formic acid^a^	Acetic acid^a^	Propionic acid^a^	Butyric acid^a^	Total VFA^b^	A/P ratio^c^	Rumen pH	Description of rumen contents
May	A	MA	0.5	b.d.^d^	b.d.	61.6	14.0	5.2	80.8	4.4	6.68	Normal^e^
May	B	MB	0.4	0.2	b.d.	64.7	12.7	2.3	79.6	5.1	6.54	Normal^e^
May	C	MC	0.4	0.2	b.d.	70.1	17.5	6.0	93.6	4.0	6.76	Normal^e^
May	D	MD	0.4	0.3	b.d.	42.7	10.4	5.2	58.2	4.1	6.85	Normal^e^
Aug	B	AB	0.9	0.3	b.d.	92.6	23.2	12.8	128.6	4.0	6.09	Normal
Aug	C	AC	0.6	0.6	b.d.	92.6	24.0	13.5	130.1	3.9	5.88	Normal
Aug	D	AD	0.8	0.4	b.d.	97.9	24.3	13.5	135.8	4.0	6.12	Normal
Aug	E	AE	0.6	0.3	b.d.	79.7	19.5	11.8	111.0	4.1	6.30	Slimy
Nov	B	NB	0.2	0.2	b.d.	73.5	17.1	13.1	103.7	4.3	6.37	Normal
Nov	C	NC	0.2	11.2	6.1	64.5	14.8	13.0	92.3	4.4	6.11	Normal
Nov	D	ND	0.2	0.2	b.d.	73.3	14.9	13.3	101.5	4.9	6.33	Normal
Nov	E	NE	b.d.	0.3	b.d.	57.0	10.9	10.2	78.2	5.2	6.65	Normal
Feb	B	FB	0.2	b.d.	b.d.	99.4	23.6	17.0	140.1	4.2	6.03	Full rumen
Feb	C	FC	0.2	b.d.	b.d.	93.8	23.0	16.1	132.9	4.1	6.02	Normal
Feb	D	FD	b.d.	b.d.	b.d.	69.0	14.7	9.7	93.4	4.7	6.82	Very full rumen
Feb	E^f^	FE	b.d.	b.d.	b.d.	59.7	14.9	7.1	81.7	4.0	6.70	Half full rumen
May +1yr	B	LB	b.d.	b.d.	b.d.	63.7	15.3	6.7	85.7	4.1	6.66	Normal^e^
May +1yr	C	LC	0.2	b.d.	b.d.	71.0	17.3	9.0	97.3	4.1	6.52	Normal^e^
May +1yr	D	LD	b.d.	b.d.	b.d.	54.4	12.3	6.2	72.9	4.4	6.78	Normal^e^
May +1yr	E	LE	0.4	0.3	b.d.	60.7	14.5	7.1	82.4	4.2	6.66	Normal^e^

Rumen contents were taken after morning milking and before new pasture was offered.

^a^ Fermentation end product in mM.

^b^Total VFA is the sum of acetic, propionic and butyric acids.

^c^The A/P is the ratio of acetic acid to propionic acid.

^d^b.d., below detection limit.

^e^Rumen was half full but this is the normal level for the cows dry period.

^f^Cow lame.

During May (autumn) the cows were “dry” (not lactating), and feed intakes were low, as were total rumen VFA concentrations (Table 3). In August (winter; early lactation) the pasture was the best quality of the five periods sampled, characterised by high ME, OMD, CP and NDF (Table 2), and the rumen contents at that time had the greatest concentrations of VFAs (average total = 126 mM), consistent with a rapid fermentation. The VFA concentrations in the rumen were lower in November when the feed quality was also lower, characterised by increased fibre fractions (ADF and NDF) and lower organic matter digestibility (OMD) of the feed (Table 2). The pasture quality improved again in February (summer) and the VFA concentrations in the rumen returned to almost the same values as measured in August. One cow (cow E) was lame at the time of sampling in February, had not eaten as much as the other cows, and had lower total VFAs compared to cows B, C and D. The rumen VFAs in cow C indicated an unusual fermentation product profile at the November (spring) sampling time, with elevated levels of lactic and formic acids, but the pH (6.11) indicated that this animal was not suffering from acidosis. The animal appeared healthy and its rumen contents appeared normal. This particular sample did not seem different with pyrosequencing analysis (see below), and so differences in the rumen VFA composition does not always indicate differences in the bacterial community structure.

### Pyrosequencing of the V1-V3 region of the 16S rRNA gene

The digest-adherent rumen bacteria were analysed using 454 Titanium pyrosequencing of the bacterial 16S rRNA gene (V1-V3). This resulted in 594,271 pyrotag reads (Table 4). After pre-processing and trimming, 327,300 sequences remained, ranging in length from 370 to 409 bases. The processing of the reads was completed by further quality filtering and removal of chimeric sequences, leaving 110,585 processed reads.

**Table 4:** Number of 16S rRNA gene sequences^a^ and taxa obtained from the digesta-adherent microbiome of all animals and sampling times^b^.

Parameters	Total	Average per animal	SD	Min	Max
Trimmed reads^c^	327300	15586	1324.4	12299	17341
Quality reads^d^	110585	5266	360.4	4607	5986
Number of OTUs^e^	1539	821	70.3	653	926
Bacterial taxa^f^	74	57	3.5	51	62

^a^Average length of sequence (nt) = 519 and covers the V1-V3 region.

^b^Number of barcoded samples = 21.

^c^Sequence reads after quality trimming in QIIME.

^d^Sequence reads after clustering with CD-HIT-OTU.

^e^Number of OTUs clustered at 96% similarity.

^f^Number of taxonomic groups, identified to the genus level if possible. The higher level taxa may contain several genera.

No bands were visible in electrophoresis gels analysing the amplification products of the no-DNA control. Even so, the reaction mixture was included in the subsequent steps through to sequencing, but no pyrosequence reads were assigned to the no-DNA control barcode. A duplicated sample was included to test the effects of the barcode on amplification efficiency of the primer pairs. This was because a few primer pairs consistently produced less PCR product, even though the amount of template DNA for each PCR was normalised. The DNA template from the worst affected sample (sample FD with barcode MID65; see S1 Table) was amplified with a second primer containing a different barcode (MID51; see S1 Table). This resulted in a greater PCR product yield from this sample, and indicated that the differences were due to the barcode and not the template DNA. The amplicons generated from this template with the two different barcoded primers were included in the pyrosequencing to assess the influence that barcode choice had on the observed sequenced community. All other barcoded primer pairs that produced lower PCR product yields were substituted for different barcoded primers that produced better PCR yields and the products from the poorly-performing pairs were not included in the sequencing. Visual inspection of summarised bacterial taxa showed that the differences in the apparent community structure in the duplicate assessments of sample FD (labelled FD.51 and FD.65 in S1 Fig) was small. UniFrac analysis, which uses phylogenetic information to calculate pairwise distances, also indicated that the duplicate samples were more similar to each other (7.11% weighted and 27.54% unweighted distances) than the average difference between any other pair of samples (11.15% weighted and 35.08% unweighted distances). So, even though there were differences in amplification efficiency, these did not appear to skew the results when the actual community structure was assessed.

The quality-filtered reads were clustered into 1539 OTUs at 96% sequence similarity, and the average number of reads, OTUs and bacterial taxa are summarised in Table 4. Comparable numbers of trimmed reads, clustered reads and OTUs were obtained for all 21 samples. An OTU clustering threshold of 96% sequence identity was chosen, as this has been shown to most accurately represent OTU clusters gained from the full length 16S rRNA sequence (clustered at 97% identity), when only the V1-V3 region of the 16S rRNA gene is used [38]. Twelve of the 21 samples had no unique OTUs and the OTUs that were unique within the remaining samples represented less than 0.3% of all the OTUs and less than 0.01% of all the reads in the processed dataset (Table 5). This demonstrated that the differences observed between the individual bacterial communities were very small. As a percentage of the total OTU number, 11.9% were found in all 21 of the samples, 21.1% were found in more than 90% of the samples, and 50.3% were found in more than 50% of the samples.

**Table 5:** Number of unique OTUs in each animal at each sampling time point^a^.

Months and code	Animal					Total^b^
	A	B	C	D	E	
**May—M**	0 (0)	3 (0.014)	0 (0)	0 (0)	n.a.	3 (0.014)
**August -A**	n.a.	1 (0.008)	0 (0)	3 (0.011)	0 (0)	4 (0.019)
**November -N**	n.a.	1 (0.005)	1 (0.008)	0 (0)	0 (0)	2(0.013)
**February—F**	n.a.	0 (0)	0 (0)	2 (0.007)	2 (0.007)	4 (0.014)
**May + 1yr—L**	n.a.	0 (0)	0 (0)	1 (0.006)	1 (0.004)	2 (0.010)
**Total**	0 (0)	5 (0.027)	1 (0.008)	6 (0.024)	3 (0.011)	

n.a., not analysed.

^a^The number of OTUs that were unique to only that sample are shown; the percentage of the total sequences this represents is shown in brackets.

^b^The total of unique OTUs and sequences found for each month or animal.

### Digesta-adherent rumen community composition

The combined processed pyrosequencing reads from all samples were classified to the most detailed taxonomic identifier available. Altogether, 74 taxa were identified. In some cases this was to the genus level, but in others to the family, order, class, phylum or domain level. The taxa identified in each sample are shown in S1 Fig. Taxonomic groups classified at levels higher than the genus level could potentially represent several genera each.

The majority of the sequences fell into the phylum Firmicutes (82.1%), followed by Bacteroidetes (11.8%) and Fibrobacteres (2.4%). Sixteen other phyla together represented another 2.2% of the sequences, and 1.5% of the sequences were unable to be assigned to a phylum.

The largest taxonomic groups were those of undefined genera in the order Clostridiales (22.9% of all sequences), followed by those that were assigned to undefined genera in the family Lachnospiraceae (12.2%) and *Butyrivibrio* (10.2%) (Table 6). Nearly two-thirds (61%) of all sequences could not be classified to the genus level. There were only ten described genera that each contained >0.5% of the total number of sequences, being (from the largest to smallest) *Butyrivibrio*, *Ruminococcus*, *Prevotella*, *Coprococcus*, *Fibrobacter*, *Clostridium*, *Mogibacterium*, *Succiniclasticum*, *Blautia* and *Shuttleworthia*. These contained, together, 36.1% of all sequences. Fifty four minor taxa each contained less than 0.5% of the total sequences, and together these 54 contained only 5% of the data (Table 7)

**Table 6:** The percentage of major bacterial taxa (>0.5% of the total) found in digesta-adherent rumen samples.

Major taxa^a^	% of total
Bacteria; Bacteroidetes; Bacteroidia; Bacteroidales; f_; g_	2.74
Bacteria; Bacteroidetes; Bacteroidia; Bacteroidales; Prevotellaceae; *Prevotella*	6.72
Bacteria; Bacteroidetes; Bacteroidia; Bacteroidales; S24-7; g_	1.54
Bacteria; Fibrobacteres; Fibrobacteria; Fibrobacterales; Fibrobacteraceae; *Fibrobacter*	2.37
Bacteria; Firmicutes; Clostridia; Clostridiales; f_; g_	22.86
Bacteria; Firmicutes; Clostridia; Clostridiales; [Mogibacteriaceae]; g_	2.25
Bacteria; Firmicutes; Clostridia; Clostridiales; [Mogibacteriaceae]; *Mogibacterium*	1.94
Bacteria; Firmicutes; Clostridia; Clostridiales; Christensenellaceae; g_	1.56
Bacteria; Firmicutes; Clostridia; Clostridiales; Clostridiaceae; *Clostridium*	2.11
Bacteria; Firmicutes; Clostridia; Clostridiales; Lachnospiraceae; g_	12.15
Bacteria; Firmicutes; Clostridia; Clostridiales; Lachnospiraceae; *Blautia*	0.83
Bacteria; Firmicutes; Clostridia; Clostridiales; Lachnospiraceae; *Butyrivibrio*	10.16
Bacteria; Firmicutes; Clostridia; Clostridiales; Lachnospiraceae; *Coprococcus*	2.60
Bacteria; Firmicutes; Clostridia; Clostridiales; Lachnospiraceae; *Shuttleworthia*	0.70
Bacteria; Firmicutes; Clostridia; Clostridiales; Lachnospiraceae; Other	3.42
Bacteria; Firmicutes; Clostridia; Clostridiales; Ruminococcaceae; g_	8.79
Bacteria; Firmicutes; Clostridia; Clostridiales; Ruminococcaceae; *Ruminococcus*	7.60
Bacteria; Firmicutes; Clostridia; Clostridiales; Veillonellaceae; *Succiniclasticum*	1.07
Bacteria; Firmicutes; Clostridia; Clostridiales; Other; Other	2.10
Unassigned; Other; Other; Other; Other; Other	1.45

^a^Taxa were defined to the lowest level possible using the greengenes release gg_13_8_otus database. Taxa labels with a blank value for the class (c_), order (o_), family (f_) or genus (g_) match a reference sequence that is poorly defined in the greengenes database. Taxa labels where one or more levels are listed as ‘other’ are due to ambiguity where the UCLUST classifier cannot distinguish between distinct taxa.

**Table 7:** Minor bacterial taxa (<0.5% of the total) found in digesta-adherent rumen samples.

Minor taxa^a^	% of total
Bacteria; Actinobacteria; Coriobacteriia; Coriobacteriales; Coriobacteriaceae; g_	0.335
Bacteria; Actinobacteria; Coriobacteriia; Coriobacteriales; Coriobacteriaceae; Other	0.004
Bacteria; Armatimonadetes; SJA-176; RB046; f_; g_	0.006
Bacteria; Bacteroidetes; Bacteroidia; Bacteroidales; [Paraprevotellaceae]; g_	0.092
Bacteria; Bacteroidetes; Bacteroidia; Bacteroidales; [Paraprevotellaceae]; CF231	0.048
Bacteria; Bacteroidetes; Bacteroidia; Bacteroidales; [Paraprevotellaceae]; YRC22	0.179
Bacteria; Bacteroidetes; Bacteroidia; Bacteroidales; [Paraprevotellaceae]; Other	0.060
Bacteria; Bacteroidetes; Bacteroidia; Bacteroidales; Bacteroidaceae; BF311	0.166
Bacteria; Bacteroidetes; Bacteroidia; Bacteroidales; BS11; g_	0.180
Bacteria; Bacteroidetes; Bacteroidia; Bacteroidales; Prevotellaceae; Other	0.022
Bacteria; Bacteroidetes; Bacteroidia; Bacteroidales; RF16; g_	0.028
Bacteria; Bacteroidetes; Bacteroidia; Bacteroidales; Other; Other	0.053
Bacteria; Chloroflexi; Anaerolineae; Anaerolineales; Anaerolinaceae; SHD-231	0.032
Bacteria; Cyanobacteria; 4C0d-2; YS2; f_; g_	0.004
Bacteria; Cyanobacteria; Chloroplast; Streptophyta; f_; g_	0.082
Bacteria; Elusimicrobia; Elusimicrobia; Elusimicrobiales; Elusimicrobiaceae; g_	0.007
Bacteria; Elusimicrobia; Elusimicrobia; Elusimicrobiales; Elusimicrobiaceae; *Elusimicrobium*	0.010
Bacteria; Elusimicrobia; Endomicrobia; o_; f_; g_	0.010
Bacteria; Firmicutes; Bacilli; Lactobacillales; Lactobacillaceae; *Lactobacillus*	0.005
Bacteria; Firmicutes; Bacilli; Lactobacillales; Streptococcaceae; *Streptococcus*	0.069
Bacteria; Firmicutes; Clostridia; Clostridiales; Clostridiaceae; g_	0.300
Bacteria; Firmicutes; Clostridia; Clostridiales; Eubacteriaceae; *Anaerofustis*	0.053
Bacteria; Firmicutes; Clostridia; Clostridiales; Lachnospiraceae; *Anaerostipes*	0.193
Bacteria; Firmicutes; Clostridia; Clostridiales; Lachnospiraceae; *Moryella*	0.248
Bacteria; Firmicutes; Clostridia; Clostridiales; Lachnospiraceae; *Pseudobutyrivibrio*	0.026
Bacteria; Firmicutes; Clostridia; Clostridiales; Ruminococcaceae; *Oscillospira*	0.139
Bacteria; Firmicutes; Clostridia; Clostridiales; Veillonellaceae; g_	0.007
Bacteria; Firmicutes; Clostridia; Clostridiales; Veillonellaceae; *Anaerovibrio*	0.045
Bacteria; Firmicutes; Clostridia; Clostridiales; Veillonellaceae; *Selenomonas*	0.167
Bacteria; Firmicutes; Clostridia; Clostridiales; Veillonellaceae; Other	0.040
Bacteria; Firmicutes; Erysipelotrichi; Erysipelotrichales; Erysipelotrichaceae;	0.028
Bacteria; Firmicutes; Erysipelotrichi; Erysipelotrichales; Erysipelotrichaceae; [Eubacterium]	0.004
Bacteria; Firmicutes; Erysipelotrichi; Erysipelotrichales; Erysipelotrichaceae; *Bulleidia*	0.165
Bacteria; Firmicutes; Erysipelotrichi; Erysipelotrichales; Erysipelotrichaceae; L7A_E11	0.268
Bacteria; Firmicutes; Erysipelotrichi; Erysipelotrichales; Erysipelotrichaceae; p-75-a5	0.071
Bacteria; Firmicutes; Erysipelotrichi; Erysipelotrichales; Erysipelotrichaceae; RFN20	0.138
Bacteria; Firmicutes; Erysipelotrichi; Erysipelotrichales; Erysipelotrichaceae; *Sharpea*	0.015
Bacteria; LD1; c_; o_; f_; g_	0.017
Bacteria; Lentisphaerae; [Lentisphaeria]; Victivallales; Victivallaceae; g_	0.023
Bacteria; Lentisphaerae; [Lentisphaeria]; Z20; R4-45B; g_	0.005
Bacteria; Planctomycetes; Planctomycetia; Pirellulales; Pirellulaceae; g_	0.017
Bacteria; Proteobacteria; Alphaproteobacteria; RF32; f_; g_	0.008
Bacteria; Proteobacteria; Alphaproteobacteria; Rickettsiales; f_; g_	0.014
Bacteria; Proteobacteria; Deltaproteobacteria; GMD14H09; f_; g_	0.031
Bacteria; Spirochaetes; Spirochaetes; Spirochaetales; Spirochaetaceae; g_	0.021
Bacteria; Spirochaetes; Spirochaetes; Spirochaetales; Spirochaetaceae; *Treponema*	0.425
Bacteria; SR1; c_; o_; f_; g_	0.361
Bacteria; Synergistetes; Synergistia; Synergistales; Dethiosulfovibrionaceae; *Pyramidobacter*	0.038
Bacteria; Tenericutes; Mollicutes; Anaeroplasmatales; Anaeroplasmataceae; g_	0.025
Bacteria; Tenericutes; Mollicutes; Anaeroplasmatales; Anaeroplasmataceae; *Anaeroplasma*	0.041
Bacteria; Tenericutes; Mollicutes; RF39; f_; g_	0.475
Bacteria; TM7; TM7-3; CW040; F16; g_	0.224
Bacteria; Verrucomicrobia; Verruco-5; LD1-PB3; f_; g_	0.005
Bacteria; WPS-2; c_; o_; f_; g_	0.018

^a^Taxa were defined to the lowest level possible using the greengenes release gg_13_8_otus database. Taxa labels with a blank value for the class (c_), order (o_), family (f_) or genus (g_) match a reference sequence that is poorly defined in the greengenes database. Taxa labels where one or more levels are listed as ‘other’ are due to ambiguity where the UCLUST classifier cannot distinguish between distinct taxa.

### Comparing sequences from different animals or sampling time points

**Fig 1:**
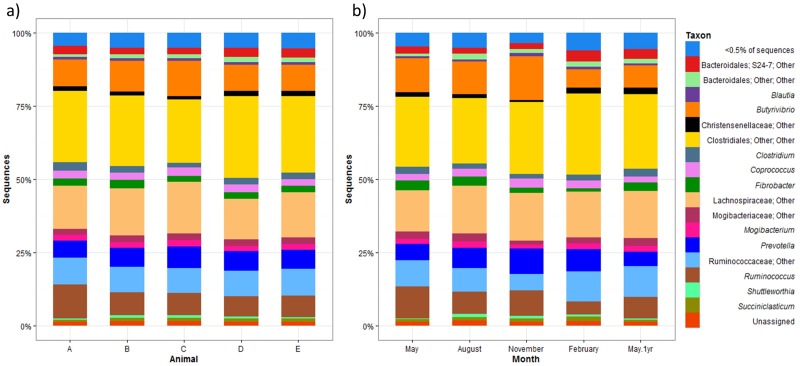
The effect of animal (a) and sampling time (b) on the bacterial genera identified from the digesta-adherent fraction of rumen contents. (a) Samples from each animal (A-E) over the course of a year. Animal A was sampled in one season and animal E was sampled in four seasons. Animals B, C and D were sampled for all five seasons. (b) Four animals were sampled once in each season (month) except for May which was sampled twice, a year apart. The key on the right shows taxa at the genus level where possible or to the lowest defined rank it could be assigned.

The relative abundances of bacterial taxa identified from the pyrosequences when the samples were grouped by animal or sample month are shown in Fig 1. The bacterial community compositions appeared similar for each animal over the five samplings, with only small differences in the abundance of the major groups (>0.5% of the total reads) and presence or absence of minor groups (<0.5% of the total reads). Because the differences between samples were small, and because not all sequences could be assigned to the genus level, the analysis was performed comparing the OTU composition of the samples, to quantify the differences between animals and sample times.

**Fig 2:**
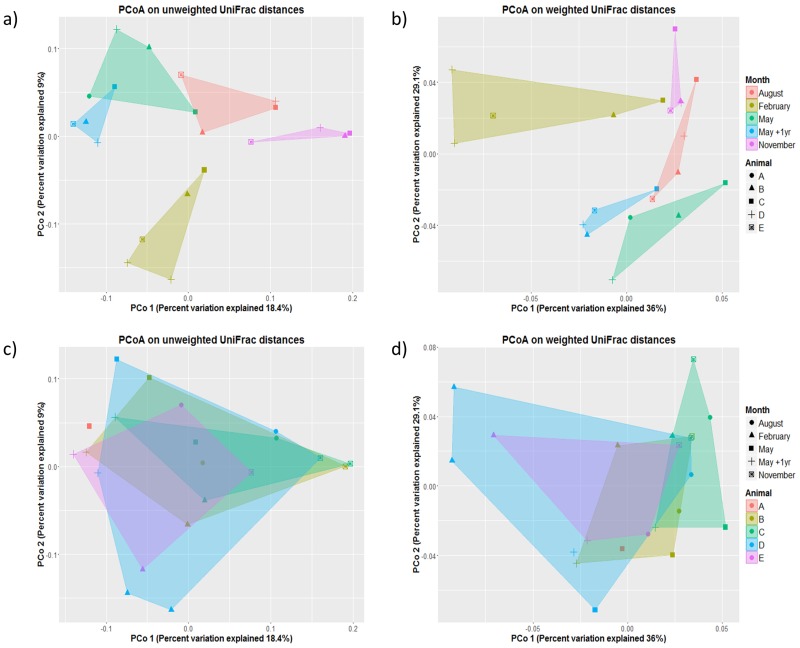
PCoA of individual samples colored by a, b) month and c, d) animal. Plots were generated using the a, c) unweighted and b, d) weighted versions of the Unifrac distance matrix.

Differences in the overall digesta-adherent bacterial community composition, determined from both weighted and unweighted UniFrac analysis, were visualised with principal coordinate analysis (PCoA) colour-coded by animal and month (Fig 2). PCoA revealed no clustering of samples by animal and a separation by month, indicated by barely overlapping clusters. Samples taken in February and November clustered completely separately from the other months on both plots. Interestingly, communities in the samples taken in May and again in May the following year clustered close to each other even though these were taken one year apart.

To examine the effects of animal-to-animal variation, summarised UniFrac distances were examined with a linear mixed model with animal as a random effect. The between-cow variance, i.e., variation between the different animals within a season, was much smaller than the within-cow variance, i.e., the variation within each animal over the seasons, with between-cow variance being 3.5% and 5.4 × 10^−7^% of the within cow-variance for weighted and unweighted UniFrac distances respectively. Anova analysis of the modelled season effects was significant (*p* < 0.001) for both the weighted and unweighted distances. The repeatability, which is the proportion of between cow-variance to the total variance, was 0.034 for weighted and 5.39 × 10^−9^ for unweighted UniFrac distances.

ANOSIM and adonis analysis on the OTUs showed significant differences in the bacterial communities from different months with the weighted (*p* = 0.001 for both analyses) and the unweighted (*p* = 0.001 for both analyses) UniFrac distance matrices. Differences in bacterial communities between different animals were not significant for the weighted (*p* = 0.82, *p* = 0.75 for ANOSIM and adonis, respectively) and the unweighted (*p* = 0.96, *p* = 0.56) UniFrac distance matrices. Interestingly, there was a significant difference between the months when the animals received silage supplementation (May and August) and the months they were only grazing on pasture (*p* = 0.001 for both analyses on weighted UniFrac distance matrices) and (*p* = 0.002 and *p* = 0.005 for ANOSIM and adonis on unweighted UniFrac distance matrices).

## Discussion

### Bacterial diversity

Our study revealed a high bacterial diversity in the rumen of animals grazing pasture under normal dairy farming practices. In total, 1539 OTUs (16S rRNA gene sequences grouped at >96% similarity) were found in the digesta-adherent fractions of all 20 rumen samples, ranging from 653 to 926 OTUs per sample. These OTU estimates are similar to those reported in other studies using 16S rRNA gene sequence analysis of rumen communities, with 613 OTUs (97% similarity) [9] and 739–946 OTUs (estimates based on rarefaction curve at 97% similarity) [6]. Most OTUs found in this study were present in more than one sample. Around 50% of the OTUs were found in more than half of the samples and 21% of the OTUs were found in more than 90% of the samples. These OTUs could represent a core microbiome for digesta-adherent bacteria in these pasture-fed dairy cows.

The prevalence of Firmicutes has been found in many other studies of rumen bacterial community composition [9, 39–41], although other studies have found Bacteroidetes to be the most abundant phylum [8, 15, 22, 42]. Together, members of Firmicutes and Bacteroidetes are always the most abundant bacteria detected in the rumen by culture-independent molecular methods. The prevalence of Firmicutes found in this study (82.1%) is one of the highest values reported for the rumen. Higher values of 90.2% and 95% of sequences assigned to Firmicutes were reported for Holstein cows on a high roughage diet and grain diet, respectively [40], but most reported values are less than 70%.

The 1539 OTUs were classified into at least 74 taxa at the genus rank or higher. This is much greater than the 23 to 27 genera reported by Kong *et al*. [9] from four cows on alfalfa or triticale diets but less than the 122 to 149 genera reported by Pitta *et al*. [6] from 14 steers fed Bermuda grass or wheat and the 180 known genera represented in the meta analysis of all 16S rRNA sequences of rumen origin from the RDP database by Kim *et al*.[39]. Some of the differences in reported genera are likely to be due to the use of different taxonomic assignment methods [43].

Together, the genera *Butyrivibrio*, *Ruminococcus*, *Prevotella*, *Coprococcus*, *Fibrobacter*, *Clostridium*, *Mogibacterium*, *Succiniclasticum*, *Blautia* and *Shuttleworthia* comprised 36.1% of the pyrosequences. Some studies have found *Prevotella* the most prevalent genus in the rumen [17, 22, 44]. In contrast, we found that *Butyrivibrio* was the largest of the known genera and comprised 10.2% of the sequences, followed by *Ruminococcus* with 7.6% of the sequences and *Prevotella* with 6.7% of the sequences. The prevalence of *Ruminococcus* and *Butyrivibrio* in the digesta-adherent fraction fits well with their known fibre-degrading capacities. The most prevalent genera here were similar to those found in other studies that used pyrosequencing [6, 9, 45]. However, undefined genera in Lachnospiraceae, Clostridiales and Ruminococcaceae account for half (49.6%) of the sequences, illustrating the need for more pure cultures of these groups to be obtained and used to improve the taxonomy of rumen bacteria. That information could be used to more accurately classify the members of the rumen bacterial community represented by the many 16S rRNA gene sequences that are not classified to the genus level, and assign functions to those bacteria based on genomic and phenotypic characterisation.

### Bacterial communities associated with different animals

Analysis of the 16S rRNA gene pyrosequences revealed that the differences between the bacterial communities in individual animals were small, and that only between 0.13% and 0.59% of the total OTUs were unique to any one animal. The differences in community composition between animals at any one time point were not statistically significant, the animal-to-animal variation was only small compared to the total variation, and PCoA analysis showed no clustering by individual animal. This is in contrast to other studies which have found substantial differences between individual animals on the same diets [6, 16, 46, 47]. The differences between animals in this study in age and milk production were not related to differences in the rumen bacterial community. Furthermore, duplicate analyses of the same sample, with two different barcoded primers, showed that the biases introduced by the choice of barcode probably contributed to some of the variation that was detected, although this was less than the variation between different samples. Overall, the invariance in the bacterial communities of different animals grazing together in this study is notable and possibly unexpected.

The sample from cow C at the November time point had an unusual fermentation end product profile compared to all the other rumen samples, with elevated levels of lactic and formic acids and low levels of acetic acid. However, this sample did not have a noticeably different bacterial community. There was only one OTU unique to this sample and it was assigned to Coriobacteriaceae. The cause of the unusual fermentation profile could not be determined, but may have been caused by a disturbance (change in activity) in the bacterial microbial community that did not affect its species composition. Because only composition was examined, and only one sample was taken from each cow at each time point, this dataset did not allow us to verify either of these theories.

### Bacterial communities associated with different time points

We showed that the composition of the pasture feed changed throughout the year and that this was associated with small but statistically significant differences in rumen bacterial community composition in different seasons. However, the effects of diet cannot be separated from potential effects caused by the production phase of the cows as these are all part of the seasonal farming system. Supplementation with pasture silage also had a small but significant effect on the rumen community. This indicates that the rumen bacterial community is not static and adapts to the modest changes in pasture diet of different qualities, and not only to major dietary changes like the well-known effects of changing diet from forage to grain. The season-specific variation in bacterial community structure was confirmed by the fact that the rumen bacterial communities in the individual animals changed more-or-less in unison, and by the close clustering of the two samples taken in the same season a year apart. This demonstrates that bacterial community structure returned to close to the community structure associated with the same season in the yearly cycle.

When samples were taken from cows over 2 lactations (2 years), there were also only small differences in the rumen bacterial communities over time [8]. These differences were proposed to be the result of cow age or cow lactation status. Those animals were fed a consistent diet of total mixed rations however the composition of the mixed rations varied over the course of the experiment, which could explain small community differences. Previous work has examined the larger changes in rumen bacterial communities when cows were transitioned from Bermuda grass hay to winter wheat [6] or fed different diets (alfalfa or triticale) [9]. Our data show that this is detectable even when diet changes are relatively small. The production herd that our animals were part of were fed a forage-based diet throughout the year.

In this study, we expected to see large differences in rumen microbial community structure over the seasons and the associated production phases of the animals. This study shows that a much larger number of animals will be needed to investigate the observed small differences in detail. Since the major bacteria did not vary to any great extent over time, it seems be reasonable to conclude that the largest part of the carbon and energy flow in the rumen of these animals was carried out by the same microbes regardless of seasonal changes in diet and animal production phase. The broad metabolic capabilities of dominant bacteria has been pointed out (48), so presumably the changes in feed over the seasons are accommodated for by this flexibility. It will be interesting to determine if this is due to changes in gene expression from within any single genotype, or if different genotypes within bacterial groups dominate at different times.

In conclusion, the work presented here revealed the bacterial community was largely similar between animals and that the small detectable differences due to the season appeared to be cyclic, returning to the composition previously seen when the feed and production phase was similar. Overall, this work demonstrates the remarkable stability of the rumen bacterial community in pasture-fed cows in a production herd over time.

## Supporting information

S1 Fig Bacterial taxa from the digesta-adherent fraction of rumen contents. Samples are identified by the month and animal they were sampled from. The first letter represents the month the sample was taken, M = May (Autumn), A = August (Winter), N = November (Spring), F = February (Summer) and L = May + 1yr (Autumn). The second letter represents the animal A, B C, D and E. Samples FD.51 and FD.65 are sequenced from the same sample (FD) with different barcodes on the forward primer. The key on the right shows taxa at the genus level where possible or to the lowest defined rank it could be assigned.
(TIF)

S1 Table Barcoded primers for multiplex pyrosequencing PCR. Barcode sequences are highlighted in bold. Samples are identified by the month and animal they were sampled from. The first letter represents the month the sample was taken, M = May (Autumn), A = August (Winter), N = November (Spring), F = February (Summer) and L = May + 1yr (Autumn). The second letter represents the animal A, B C, D and E.
(DOCX)
