# Supplementary figures and images for: Seasonal changes in the digesta-adherent rumen bacterial communities of dairy cattle grazing pasture
Source: PLoS One. 2017 Mar 15;12(3):e0173819. doi: 10.1371/journal.pone.0173819 (PMC5351972; doi:10.1371/journal.pone.0173819)

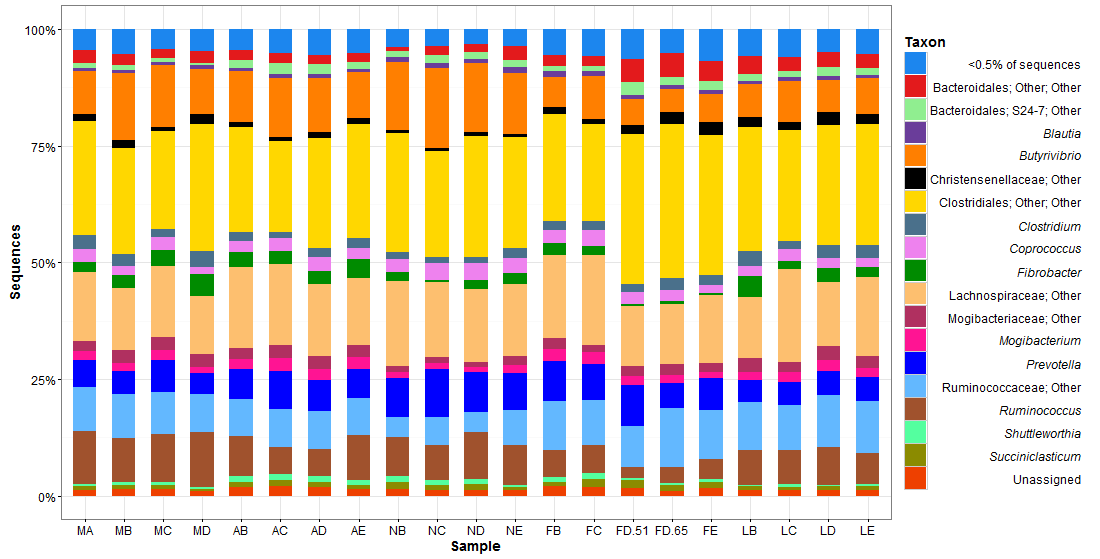

Supplement: S1 Fig — Samples are identified by the month and animal they were sampled from. The first letter represents the month the sample was taken, M = May (Autumn), A = August (Winter), N = November (Spring), F = February (Summer) and L = May + 1yr (Autumn). The second letter represents the animal A, B C, D and E. Samples FD.51 and FD.65 are sequenced from the same sample (FD) with different barcodes on the forward primer. The key on the right shows taxa at the genus level where possible or to the lowest defined rank it could be assigned. (TIF) [file pone.0173819.s001.tif]
