# Supplementary material for: Seasonal changes in the digesta-adherent rumen bacterial communities of dairy cattle grazing pasture
Source: PLoS One. 2017 Mar 15;12(3):e0173819. doi: 10.1371/journal.pone.0173819 (PMC5351972; doi:10.1371/journal.pone.0173819)
Supplement: S1 Table — Barcode sequences are highlighted in bold. Samples are identified by the month and animal they were sampled from. The first letter represents the month the sample was taken, M = May (Autumn), A = August (Winter), N = November (Spring), F = February (Summer) and L = May + 1yr (Autumn). The second letter represents the animal A, B C, D and E. (DOCX) [file pone.0173819.s002.docx]

Table S1. Barcoded primers for multiplex pyrosequencing PCR

| Primer | Sample | Primer sequence (5’ - 3’) |
| --- | --- | --- |
| Afd1*MID55 | AB | CGT ATC GCC TCC CTC GCG CCA TCA G**AT** **CTA CGC** **TG**G AGT TTG ATC MTG GCT CAG |
| Afd1*MID56 | AC | CGT ATC GCC TCC CTC GCG CCA TCA G**CT** **CGC ATA** **CG**G AGT TTG ATC MTG GCT CAG |
| Afd1*MID57 | AD | CGT ATC GCC TCC CTC GCG CCA TCA G**AC** **AGA CAC** **GT**G AGT TTG ATC MTG GCT CAG |
| Afd1*MID58 | AE | CGT ATC GCC TCC CTC GCG CCA TCA G**CA** **CGA TCT** **AC**G AGT TTG ATC MTG GCT CAG |
| Afd1*MID59 | NB | CGT ATC GCC TCC CTC GCG CCA TCA G**AG** **CGC TCA** **GT**G AGT TTG ATC MTG GCT CAG |
| Afd1*MID60 | NC | CGT ATC GCC TCC CTC GCG CCA TCA G**CG** **CTA GTG** **TA**G AGT TTG ATC MTG GCT CAG |
| Afd1*MID61 | ND | CGT ATC GCC TCC CTC GCG CCA TCA G**GT** **AGT ACA** **TG**G AGT TTG ATC MTG GCT CAG |
| Afd1*MID62 | NE | CGT ATC GCC TCC CTC GCG CCA TCA G**TG** **AGT GTC** **AC**G AGT TTG ATC MTG GCT CAG |
| Afd1*MID63 | FB | CGT ATC GCC TCC CTC GCG CCA TCA G**GA** **GCA CTA** **GC**G AGT TTG ATC MTG GCT CAG |
| Afd1*MID64 | FC | CGT ATC GCC TCC CTC GCG CCA TCA G**TA** **GCA CGC** **GA**G AGT TTG ATC MTG GCT CAG |
| Afd1*MID65 | FD | CGT ATC GCC TCC CTC GCG CCA TCA G**GC** **TGA GTC** **AC**G AGT TTG ATC MTG GCT CAG |
| Afd1*MID66 | FE | CGT ATC GCC TCC CTC GCG CCA TCA G**TC** **TGA CAC** **TC**G AGT TTG ATC MTG GCT CAG |
| Afd1*MID67 | LB | CGT ATC GCC TCC CTC GCG CCA TCA G**GT** **CTG CTC** **AG**G AGT TTG ATC MTG GCT CAG |
| Afd1*MID68 | LC | CGT ATC GCC TCC CTC GCG CCA TCA G**TG** **CGA CTG** **AT**G AGT TTG ATC MTG GCT CAG |
| Afd1*MID69 | LD | CGT ATC GCC TCC CTC GCG CCA TCA G**GA** **CGA TGC** **AT**G AGT TTG ATC MTG GCT CAG |
| Afd1*MID70 | LE | CGT ATC GCC TCC CTC GCG CCA TCA G**TA** **CGC AGC** **TG**G AGT TTG ATC MTG GCT CAG |
| Afd1*MID71 | MA | CGT ATC GCC TCC CTC GCG CCA TCA G**GC** **GTG TAT** **GC**G AGT TTG ATC MTG GCT CAG |
| Afd1*MID72 | MB | CGT ATC GCC TCC CTC GCG CCA TCA G**TC** **GCG CTC** **TA**G AGT TTG ATC MTG GCT CAG |
| Afd1*MID73 | MC | CGT ATC GCC TCC CTC GCG CCA TCA G**AC** **GTA CTC** **AC**G AGT TTG ATC MTG GCT CAG |
| Afd1*MID74 | MD | CGT ATC GCC TCC CTC GCG CCA TCA G**CA** **GTG CTC** **TC**G AGT TTG ATC MTG GCT CAG |
| Afd1*MID54 | -ve | CGT ATC GCC TCC CTC GCG CCA TCA G**AG** **ATG ATA** **GC**G AGT TTG ATC MTG GCT CAG |
| Afd1*MID51 | FD | CGT ATC GCC TCC CTC GCG CCA TCA G**AC** **GCT ATC** **GA**G AGT TTG ATC MTG GCT CAG |
| B514*R |  | CTA TGC GCC TTG CCA GCC CGC TCA GCC GCG GCK GCT GGC AC |

Barcode sequences are highlighted in bold. Samples are identified by the month and animal they were sampled from. The first letter represents the month the sample was taken, M = May (Autumn), A = August (Winter), N = November (Spring), F = February (Summer) and L = May + 1yr (Autumn). The second letter represents the animal A, B C, D and E.
